# Structural quality of health facilities to provide family planning services in Ethiopia: Evidence from the 2021–22 Ethiopia Service Provision Assessment survey

**DOI:** 10.1371/journal.pgph.0005377

**Published:** 2025-12-02

**Authors:** Tewachew Muche Liyeh, Angela Dawson, Abela Mahimbo, Andrew Hayen

**Affiliations:** 1 College of Health Sciences, Debre Tabor University, Debre Tabor, Ethiopia; 2 School of Public Health, Faculty of Health, University of Technology Sydney, Sydney, Australia; School of Public Health, College of Health Science, Addis Ababa University, ETHIOPIA

## Abstract

The structural quality of family planning services reflects the capacity of health facilities to deliver effective care through adequate infrastructure, supplies, commodities, and trained personnel. While some evidence exists, recent national-level estimates of structural quality in Ethiopia remain limited. This study addresses this gap by assessing the overall structural quality of family planning services and the factors influencing it. Using data from the 2021–22 Ethiopia Service Provision Assessment survey, we assessed the structural quality across four domains: availability of trained staff, family planning guidelines, contraceptives, and equipment and supplies, aggregated into a composite score ranging from 0 to 100%. A survey-weighted multiple linear regression model was applied to assess associations between structural quality and facility characteristics, management factors, and regional variation. Among 1,102 health facilities, the estimated overall structural quality score for family planning services was 42.4% (95% CI: 41.2% to 43.6%). The availability of family planning guidelines, trained staff, equipment and supplies, and contraceptives was 55.9%, 24.1%, 45.6%, and 44.1%, respectively. Structural quality was 18.4% lower in private clinics (95% CI: –25.1% to –11.7%) and 17.0% lower in health posts (95% CI: –24.3% to –9.6%) compared to hospitals. Compared to metropolitan regions, the score was 12.4% lower in Gambela (95% CI: –20.3% to –4.6%) and 13.5% higher in Benishangul Gumuz (95% CI: 4.2% to 22.7%). Facilities that received external supervision in the past six months had 4.9% (95% CI: 0.1% to 9.7%) higher structural quality than those without supervision. Overall, the structural quality of facilities providing family planning services in Ethiopia remains suboptimal, with notable variations by facility type, region, and supervision status. Strengthening facility readiness, especially in underperforming settings, and ensuring regular supervision are essential to improving the capacity of facilities to deliver effective family planning services and advance progress toward Family Planning 2030 commitments.

## Introduction

Family planning (FP) is a cost-effective public health intervention that reduces maternal morbidity and mortality through improved access to contraception [1–3]. Contraceptive use enhances maternal and newborn health by preventing unintended pregnancies and reducing the risks of adverse outcomes during pregnancy, childbirth, and the postpartum period [4,5]. Ensuring access to appropriate, affordable, safe, and effective modern contraceptive methods is essential to meet the high demand for family planning and addressing women’s sexual and reproductive health needs [6,7].

In 2021, 77% of women aged 15–49 years globally were able to meet their family planning needs through the use of modern contraceptive methods [8]. However, regional disparities persist. While over 80% of demand was satisfied in Australia and New Zealand, Eastern and South-Eastern Asia, Europe, North America, and Latin America and the Caribbean, the rates were significantly lower in sub-Saharan Africa (56%) and Oceania, excluding Australia and New Zealand (52%) [9]. One contributing factor to the lower coverage could be the limited availability of contraceptives in health facilities, which constrains women’s access to family planning services [10,11].

The structural quality, or readiness, of family planning services refers to health facilities’ capacity to deliver services effectively, including improved physical infrastructure, a well-trained workforce, and an adequate supply of essential resources and materials [12]. Strengthening health facilities’ readiness for providing FP services through trained staff, guidelines, equipment and supplies, medicines, and commodities can significantly improve contraceptive uptake and reduce unmet need [13,14].

Despite ongoing efforts, low contraceptive use [15] and high unmet need for FP [16] remain persistent challenges in Ethiopia. To address these challenges, the Ethiopian Ministry of Health has expanded access to family planning services by strengthening its community-based Health Extension Program [17]. Additionally, the health sector has also introduced a national guideline to integrate family planning services into primary health care that includes antenatal, intrapartum, and postnatal care, child health, immunization, and the management and prevention of human immunodeficiency virus (HIV) [18]. Furthermore, the government has endorsed the Family Planning 2030 (FP2030) commitment, a global initiative aimed at ensuring universal access to quality contraceptive services and reproductive healthcare [19].

Healthcare facilities in Ethiopia frequently face challenges in meeting the growing demand for family planning services due to capacity constraints [20,21]. Assessing their readiness is essential for improving family planning programs. Prior evidence on structural quality exists, but comprehensive and recent national-level evidence and its determinants in Ethiopia are limited. Therefore, this study assessed the overall structural quality of family planning services and factors influencing it in Ethiopia using the 2021–22 Ethiopia Service Provision Assessment (ESPA) survey [22]. The findings are essential for policymakers to identify gaps and make informed decisions to improve structural quality, increase service use, and reduce unmet needs.

## Materials and methods

### Study design and setting

This cross-sectional study was conducted in Ethiopia, the second-largest and second-most populous country in Africa [23]. During the study period, the country comprised ten regional states: Amhara, Afar, Benishangul-Gumuz, Gambela, Harari, Oromia, Sidama, Somali, Southern Nations Nationalities and Peoples’ Region (SNNPR), and Tigray—as well as two city administrations: Addis Ababa and Dire Dawa. Although the ESPA 2021 classifies SNNPR as one region, subsequent administrative changes divided it into SNNPR, Southwest Ethiopia, and Central Ethiopia regions. Our analysis reflects the survey’s original classification. As of 2023, about 23% of the Ethiopian population resides in urban areas [24]. The public healthcare system is organized into three tiers: primary, secondary, and tertiary, with family planning services available at all levels [22].

### Source of data and data collection instruments

We used data from the 2021–22 Ethiopia Service Provision Assessment (ESPA) survey. Data were collected from August 11, 2021, to February 4, 2022, and covered all hospitals and a sample of health centres, clinics, health posts, and private health facilities across the country, excluding the Tigray region, for security reasons. A facility inventory questionnaire was used to assess the functional capacity of health facilities to provide services that meet acceptable standards. The ESPA dataset includes comprehensive information on health facility characteristics as well as the availability and readiness of services, including family planning. Detailed descriptions of data collection methods, recruitment methods, and quality assurance procedures for ESPA-2021–22 are available elsewhere [22]. The datasets are publicly accessible through the Demographic and Health Survey Program website (https://dhsprogram.com). We accessed the data for research purposes on July 1, 2024.

### Sampling and sample size determination

The 2021–22 ESPA survey used a master list of 25,711 functioning health facilities in Ethiopia. The survey used a combination of census and stratified sampling techniques to select health facilities. Health facilities within each region were first stratified by type (hospitals, health centres, health posts, and clinics). A representative sample of health centres, health posts, and clinics was then selected. All hospitals (government and private) were included in the sample due to their small number and critical role in the health system. Of the 1,407 health facilities selected for the study, 249 were excluded due to permanent closure, inaccessibility, or security concerns, and ESPA data were collected from 1,158 facilities. Family planning data were collected explicitly from 1,044 (a weighted sample of 1,102) health facilities that provided FP services in Ethiopia. Sampling weights were calculated using the inverse probability of selection within each stratum.

### Variables and measurements

The outcome variable was the structural quality (readiness) of family planning services, measured using the Service Availability and Readiness Assessment (SARA) service-specific readiness indicators, across four domains: availability of trained staff, family planning guidelines, equipment and supplies, and contraceptives [12]. Facility type, regions, facility setting, external supervision, established quality structures, and routine management meetings were included as explanatory variables. We classified the Harari region, Addis Ababa, and Dire Dawa city administrations as metropolitan areas (Table 1).

**Table 1:** Measurement of variables used to analyse disparities in the structural quality of health facilities to provide family planning services in Ethiopia.

Variables	Scoring method	Details
Trained staff	Binary (0 or 1)	A score of ‘1’ was assigned if the facility had at least one provider trained in family planning within the past 24 months; otherwise, a score of ‘0’.
Family planning (FP) guidelines	Binary (0 or 1)	A score of ‘1’ was assigned if the facility had at least one FP guideline; otherwise, a score of ‘0’ was assigned.
Contraceptives	Continuous (0–1)	Determined based on the availability of ten modern family planning methods: combination oral pills, progestin-only oral pills, progestin-only injectables, male condoms, female condoms, intrauterine devices (IUDs), implants, tubal ligation, vasectomy, and emergency contraception. The average score was calculated by dividing the number of all available methods by ten.
Equipment and supplies:	Continuous (0–1)	measured using the availability of seven components: a blood pressure (BP) apparatus, an examination light, an examination bed, samples of FP methods, a demonstration model for condom use, a pelvic model for demonstrating IUD, and visual aids. The average score was calculated by dividing the number of available components by seven.
The structural quality of FP services	Composite score (0–100)	Calculated by averaging the scores across all domains (trained staff, FP guideline, contraceptives, and equipment and supplies) multiplied by 100, yielding the final score of 0–100.
Facility type	Categorical	Hospitals, Health centres, Health posts, and Clinics
Facility Setting	Categorical	Urban, Rural
Location	Categorical	Administrative regions: Amhara, Afar, Benishangul-Gumuz, Gambela, Oromia, Sidama, Somali, Southern Nations Nationalities and Peoples’ Region (SNNPR), and Metropolitan regions.
External supervision	Binary (0 or 1)	Score = 1 if the facility had external supervision within the past six months; otherwise, 0.
Established quality structure	Binary (0 or 1)	Score = 1 if the facility had established a quality improvement structure; otherwise, 0.
Routine management meeting	Binary (0 or 1)	Score = 1 if the facility conducted routine monthly or more often management/administrative meetings; otherwise, 0.

### Data processing and management

After accessing the ESPA 2021–22 datasets in Stata format, we restricted the sample to facilities reported as providing family planning services. Variables used in this study were extracted from the facility inventory questionnaire, and the raw data were checked for completeness. We recoded variables into consistent formats, and data processing was done using Stata 18 (StataCorp, College Station, TX, USA).

### Data analyses

We used frequencies, percentages, and means to summarise the characteristics of variables as appropriate. We applied weights as specified in the ESPA survey to address the sampling and non-response rate and adjust the data. We used the Stata “*svy*” suite of commands to reflect the sampling methods. We used a multiple linear regression model to assess the association between the overall structural quality of family planning services and the facility type, region, and facility setting. Wald test P values were used to assess the joint significance of each group of predictors in the model.

### Ethical approval

We obtained ethical approval from the Human Research Ethics Committee of the University of Technology Sydney (UTS HREC REF NO. ETH23–7996) and the institutional review board of the Ethiopian Public Health Institute (EPHI-IRB-544–2023). Informed written or verbal consent was not required, as the study used secondary data from a publicly available, anonymised online database.

## Results

The analysis included 1,102 health facilities. The majority (79.5%) were located in rural areas. More than two-thirds (67.2%) were health posts, while a small proportion (2%) were hospitals. Regarding regional distribution, approximately 38% of the facilities were located in Oromia, 23% in SNNP, and 22% in the Amhara region (Table 2).

**Table 2:** Distribution of health facilities by type, region, and setting in Ethiopia, 2021-22.

Variables	Categories	Types of health institutions				Total n (%)
		Hospital n (%)	Health centre n (%)	Health post n (%)	Private clinic n (%)	
Setting						
	Urban	5 (22.7)	56 (31.0)	58 (7.8)	107 (67.3)	226 (20.5)
	Rural	17 (77.3)	125 (69.0)	682 (92.2)	52 (32.7)	876 (79.5)
Region						
	Metropolitan	3 (13.6)	7 (3.9)	4 (0.5)	20 (12.5)	34 (3.0)
	Gambela	0 (0.0)	2 (1.1)	6 (0.8)	6 (3.8)	14 (1.3)
	Afar	0 (0.0)	4 (2.2)	11 (1.5)	2 (1.2)	17 (1.5)
	Amhara	5 (22.7)	41 (22.6)	155 (21.0)	41 (25.8)	242 (22.0)
	Oromia	7 (31.8)	71 (39.0)	278 (37.6)	64 (40.3)	420 (38.0)
	Somali	1 (4.6)	11 (6.0)	47 (6.4)	2 (1.2)	61 (5.5)
	Benishangul Gumuz	0 (0.0)	2 (1.1)	15 (2.0)	3 (1.9)	20 (1.8)
	SNNPR	5 (22.7)	35 (19.0)	192 (26.0)	19 (11.9)	251 (23.0)
	Sidama	1 (4.6)	8 (4.4)	32 (4.3)	2 (1.2)	43 (4.0)
	Total	22	181	740	159	1,102 (100)
External supervision						
	Yes	18 (82.6)	125 (68.9)	672 (90.8)	86 (54.0)	901 (81.7)
	No	4 (17.4)	56 (31.1)	68 (9.2)	73 (46.0)	201 (18.3)
Quality structure						
	Yes	18 (81.3)	125 (69.4)	59 (8.0)	28 (17.9)	231 (21.0)
	No	4 (18.7)	55 (30.6)	681 (92.0)	131 (82.1)	871 (79.0)
Monthly management meeting						
	Yes	21 (94.0)	166 (92.0)	230 (31.0)	51 (32.0)	467 (42.4)
	No	1 (6.0)	15 (8.0)	510 (69.0)	108 (68.0)	635 (57.6)

### Structural quality of family planning services

**Fig 1:**
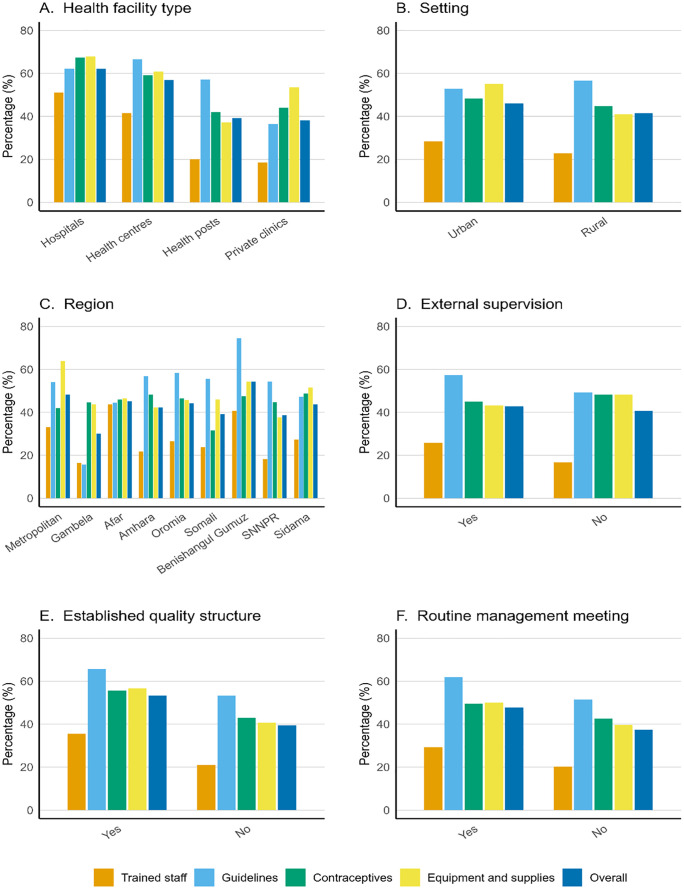
Quality of health facilities providing family planning services in Ethiopia, 2021–22.

The availability of national family planning guidelines, trained staff, equipment and supplies, and contraceptives was 55.9%, 24.1%, 45.6%, and 44.1%, respectively. Overall, the mean structural quality score of family planning services was 42.4% (95% CI: 41.2% to 43.6%). Hospitals achieved the highest mean score (62.1%), while private clinics scored the lowest (38.1%) (Fig 1A). Urban facilities scored slightly higher than rural facilities (46.1% vs 41.4%) (Fig 1B). Regionally, Benishangul Gumuz (54.2%) and metropolitan areas (48.2%) scored relatively better, whereas Gambela (30.1%), Somali (39.2%), and SNNPR (38.7%) had the lowest scores (Fig 1C). Facilities with external supervision (42.8%), a quality improvement structure (53.4%), and those that held routine management meetings (47.7%) achieved better scores than those without (Fig 1D–1F). Oral contraceptives, injectables, and implants were the most commonly available methods (S1 Table).

Mean structural quality scores are displayed by facility type, urban/rural setting, region, and selected management and supervision factors.

### Factors affecting the structural quality of family planning services

We identified factors affecting the structural quality of family planning across different facility characteristics, management practices, and geographic factors. Compared to hospitals, the structural quality score was 18.4% lower at private clinics (95% CI: –25.1% to –11.7%) and 17% lower at health posts (95% CI: –24.3% to –9.6%). Additionally, a significant difference was observed across regions. Compared to metropolitan regions, the Gambela region had a 12.4% lower structural quality score (95% CI: –20.3% to –4.6%), while the Benishangul Gumuz region had a 13.5% higher score (95% CI: 4.2% to 22.7%). Facilities with external supervision in the past six months had 4.9% (95% CI: 0.1% to 9.7%) higher structural quality score than those without supervision. We did not observe a difference in the structural quality score between urban and rural settings (Table 3).

**Table 3:** Factors associated with the structural quality of family planning services in Ethiopia, 2021-22.

Facility characteristics		Unadjusted Coefficient (95% CI)	Adjusted coefficient (95% CI)	Wald test p-value
Facility type				<0.001
	Hospitals (ref)	–	–	
	Health posts	–23.0 (–28.9, –17.1)	–17.0 (–24.3, –9.6)	
	Health centres	–5.1 (–11.2, 1.1)	–3.1 (–8.3, 2.1)	
	Private clinics	–23.4 (–30.3, –17.7)	–18.4 (–25.1, –11.7)	
Setting				0.415
	Urban (ref)	–	–	
	Rural	−4.8 (−9.30, −0.30)	−2.2 (−7.4, 3.0)	
Region				<0.001
	Metropolitan(ref)	–	–	
	Gambela	−18.1 (−26.7, −9.6)	−12.4 (−20.3, −4.6)	
	Afar	−3.0 (−14.2, 8.1)	1.5 (−9.7, 12.8)	
	Amhara	−5.9 (−14.2, 2.5)	−0.3 (−7.9, 7.4)	
	Oromia	−3.9 (−12.2,4.4)	0.7 (−6.8, 8.2)	
	Somali	−9.0 (−19.8, 1.8)	−3.1 (−14.2, 7.9)	
	Benishangul Gumuz	6.0 (−3.2, 15.2)	13.5 (4.2, 22.7)	
	SNNPR	−9.5 (−17.6, −1.3)	−4.5 (−12.2, 3.3)	
	Sidama	−4.5 (−13.3, 4.2)	−2.1 (−10.5, 6.3)	
External supervision				0.046
	No (Ref)	–	–	
	Yes	2.2 (−2.8, 7.2)	4.9 (0.1, 9.7)	
Established quality structure				0.090
	No (Ref)	–	–	
	Yes	13.9 (9.1, 18.8)	4.9 (−0.8, 10.5)	
Routine management meeting				0.393
	No (Ref)	–	–	
	Yes	9.2 (4.9, 13.5)	2.3 (−2.9, 7.4)	
				

## Discussion

We found that the overall structural quality of family planning services in Ethiopian health facilities was suboptimal, with substantial variations across facility type, region, and supervision status over the past six months. Only about one in four facilities had providers who had received in-service training in family planning within the past two years, and just over half reported having national guidelines. On average, facilities offered fewer than half of the essential modern contraceptive methods, and the availability of basic equipment and supplies was similarly limited. Collectively, these gaps highlight persistent weaknesses in the readiness of the health facilities to deliver comprehensive, high-quality family planning services.

The low overall structural quality score highlights substantial gaps in the availability of essential human and physical resources for family planning services. Similar findings have been reported in previous studies across Africa [14,25], which documented widespread inadequacies in structural quality. Such deficits suggest that many facilities may lack the infrastructure, trained personnel, equipment, or supplies required to deliver and sustain high-quality care. Evidence indicates that facilities with better service readiness are more likely to improve contraceptive use. For example, a study in Ethiopia [26] and in Indonesia [27] found that women living closer to health facilities with higher structural quality were more likely to use modern contraceptives compared to those near low-quality facilities. These findings underscore the critical role of strengthening facility readiness in Ethiopia, where unmet need remains high [16] and FP service coverage is low [15].

Although the Federal Ministry of Health of Ethiopia has implemented free in-service training programs for over two decades and more recently adopted a structured on-the-job training approach, our findings reveal a critical gap: only one in four facilities had providers with recent FP training. This proportion is notably lower than the 44% reported in the 2014 ESPA survey, suggesting a declining emphasis on staff capacity development for FP providers over time [28]. The absence of continuous training may hinder the delivery of high-quality, evidence-based family planning services, potentially compromising the support available to individuals seeking such services [29]. Therefore, it should be a priority to scale up in-service training, ensure that it is continuously updated, and monitor its effectiveness. Post-training supervision and improved working conditions are essential to support trained staff in delivering effective services [30].

The absence of national family planning guidelines in nearly half of the health facilities presents another barrier to consistent service quality. Guidelines are essential for standardising service delivery and ensuring adherence to evidence-based practices. Without them, care may become inconsistent and fall short of national or global standards [31]. A study from Kenya [32] demonstrated that facilities with updated guidelines were more likely to deliver high-quality family planning services. Although our findings show a modest improvement compared with the ESPA 2014 survey, substantial gaps remain. Ensuring that every facility has updated guidelines, coupled with provider orientation, supportive supervision, and regular monitoring, will be essential for their use and impact [33].

Contraceptive methods, availability, and supply of essential equipment were also limited. Facilities lacking a comprehensive method mix and necessary equipment may fail to meet clients’ needs, contributing to dissatisfaction or discontinuation [34,35]. While injectables and implants are the most commonly used contraceptives in Ethiopia [22], our findings revealed that 12.5% of health facilities lacked injectables and 27% lacked implants. This shortage constrains client choice and undermines providers’ ability to deliver comprehensive counselling. Evidence showed that inadequate contraceptive availability was associated with a higher unmet need for family planning [36]. Expanding a broad method mix and ensuring consistent equipment supply across facilities are therefore critical for promoting informed choice and helping individuals achieve fertility intentions [37].

Significant disparities in overall structural quality score were observed across different facility types. Private clinics and health posts had notably lower scores compared to hospitals, consistent with a previous study in Ethiopia [38]. This discrepancy may be attributed to differences in funding, infrastructure, and resource allocation. Health posts, which primarily serve remote and underserved communities, often face logistical constraints that hinder the consistent availability of essential resources. Private clinics may prioritise more profitable services and, being less integrated with the government supply chain, often face weaker regulatory oversight, which limits their investments in staff training and FP commodities [39]. These findings highlight the need for targeted investments and capacity-building efforts to improve service readiness in private clinics and health posts. In the Ethiopian context—characterised by a high fertility rate and substantial unmet needs [40], particularly in the rural areas — it is critical to strengthen the readiness and availability at health posts to ensure accessible and equitable family planning services for remote populations. However, this pattern may not be universal. For example, a study in the Democratic Republic of the Congo reported that public facilities had a lower structural quality than private ones [25]. In this context, public health facilities frequently provide FP services free of charge, which can result in overburdened systems and recurrent stockouts of essential supplies.

This study also identified notable differences in structural quality levels across regions, highlighting an unequal distribution of resources needed for region-specific strategies to improve family planning services. The Benishangul Gumuz region had a higher score than the metropolitan areas, likely reflecting its smaller, predominantly public health system—which may have benefited from concentrated donor support and closer oversight—and achieves a relatively higher score. In contrast, metropolitan areas, dominated by private clinics, consistently underperformed on structural quality indicators in this study, resulting in an overall low score. On the other hand, lower quality scores were observed in the Gambela region. The Gambela region, one of Ethiopia’s most remote and underserved areas [41], faces challenges such as poor infrastructure, including roads and transportation, which could hinder the movement of healthcare providers and the delivery of supplies, including contraceptives. These findings underscore the importance of tailored interventions to ensure equitable access to quality FP services across all regions. One such approach is mobile outreach services, which allow for the strategic and flexible deployment of healthcare providers, family planning commodities, supplies, equipment, vehicles, and infrastructure to underserved areas, ensuring timely and demand-responsive service delivery [42,43].

Consistent with findings from low- and middle-income countries [13,44], facilities with external supervision in the past six months demonstrated higher structural quality than those without. Regular supervision can reinforce service standards, identify gaps in supplies and infrastructure, and facilitate corrective actions. Supervision also provides opportunities for on-site coaching and strengthens accountability. These findings highlight the importance of maintaining structured, supportive supervision as a strategy for improving service readiness [45,46].

Finally, in line with findings from Nepal [47], we found no significant differences in family planning service readiness scores between urban and rural areas. This suggests that low readiness is a common issue across both settings, and improvement efforts should be equally distributed between urban and rural facilities. The absence of an urban-rural gap may partly reflect the influence of Ethiopia’s health extension programme and mobile health workers in rural areas, which may have contributed to narrowing the gap between urban and rural facilities.

### Strengths and limitations

A key strength of this study is its use of national data from the 2021–22 Ethiopian Service Provision Assessment (ESPA) survey. In addition, using multiple indicators enabled a thorough evaluation of this quality. Nevertheless, several limitations should be acknowledged. First, although we used the Service Availability and Readiness Assessment (SARA) indicators to assess the structural quality score, it may oversimplify service capacity. For instance, the presence of a single trained provider may be insufficient for larger health facilities that serve high patient volumes, requiring multiple trained staff. Moreover, while the provider may be trained in a specific aspect of care, the facility may still lack other essential skills that necessitate ongoing training and updates. Second, this study’s focus solely on structural quality does not evaluate the process and outcome aspects of care, which may limit the overall comprehensiveness of the quality assessment. Third, the data were based on facility reports, which may be subject to reporting bias. Finally, excluding the Tigray region for security reasons may limit the generalisability of findings to the entire country.

## Conclusion

The structural quality of health facilities providing family planning services in Ethiopia remains inadequate, with substantial variations by facility type, region, and supervision status. Many facilities lacked recently trained staff, up-to-date guidelines, essential equipment, and a comprehensive mix of contraceptive methods, limiting their readiness to deliver high-quality care. Sustained efforts are essential to strengthen readiness, improve contraceptive access, ensure informed choice, and reduce unmet need for family planning.

### Recommendations

Given the overall low structural quality score and variations across facility characteristics, geographical locations, and oversight systems, strengthening FP readiness requires a coordinated, multi-level intervention. At the national level, the Federal Ministry of Health should prioritise scaling up in-service training, ensure universal access to updated guidelines, and strengthen the supply chain to guarantee a full contraceptive method mix. Regional health bureaus should target underperforming areas and facility types, particularly health posts and private clinics, while maintaining consistent supervisory systems. At the facility level, managers should monitor staff training needs and ensure the consistent availability of contraceptives and essential equipment to improve service readiness and quality.

## Supporting information

S1 Table Percentage of health facilities offering modern contraceptive methods in Ethiopia, 2021–22. (DOCX)
