# Supplementary material for: Structural quality of health facilities to provide family planning services in Ethiopia: Evidence from the 2021–22 Ethiopia Service Provision Assessment survey
Source: PLOS Glob Public Health. 2025 Dec 2;5(12):e0005377. doi: 10.1371/journal.pgph.0005377 (PMC12671807; doi:10.1371/journal.pgph.0005377)
Supplement: S1 Table — (DOCX) [file pgph.0005377.s001.docx]

| Contraceptive methods | % of health facilities offering the services |
| --- | --- |
| COC | 88.1 |
| Injectable | 87.5 |
| Implant | 73.4 |
| Male condom | 70.7 |
| POP | 65.5 |
| Emergency contraceptive pills | 49.3 |
| IUD | 17.1 |
| Female condom | 2.4 |
| Vasectomy | 0.01 |
| Tubal ligation | 0.01 |

S1 table: Percentage of health facilities offering modern contraceptive methods in Ethiopia.
